# Serious Suicide Attempts: Systematic Review of Psychological Risk Factors

**DOI:** 10.3389/fpsyt.2018.00056

**Published:** 2018-03-07

**Authors:** Yari Gvion, Yossi Levi-Belz

**Affiliations:** ^1^Bar-Ilan University, Ramat Gan, Israel; ^2^Ruppin Academic Center, Emek Hefer, Israel

**Keywords:** suicide, medical lethality, suicide intent, mental pain, decision making, interpersonal, systematic review

## Abstract

**Background:**

One of the main obstacles in studying suicide risk factors is the difference between cases in which the individual died by suicide and those in which the individual engaged in suicidal behavior. A promising strategy that overcomes this obstacle is the study of survivors of *serious suicide attempt* (SSA), i.e., an attempt that would have been lethal had it not been for the provision of rapid and effective emergency treatment. Serious suicide attempters are epidemiologically very much like those who died by suicide, and thus may serve as valid proxies for studying suicides. This paper aims to define the specific risk factors for SSAs by conducting a qualitative data synthesis of existing studies.

**Methods:**

Following Preferred Reporting Items for Systematic Reviews and Meta-Analyses guidelines, we conducted a systematic search of the literature in PubMed, ProQuest, and Psychlit electronic research-literature databases. Search terms were “serious” “OR” “near lethal,” combined with the Boolean “AND” operator with “suicide*.” In addition, we performed a manual search on Google Scholar for further studies not yet identified.

**Results:**

The preliminary search identified 683 citations. A total of 39 research reports that met the predefined criteria were analyzed. Mental pain, communication difficulties, decision-making impulsivity, and aggression, as well as several demographic variables, were found to be major risk factors for SSAs.

**Limitations:**

We found a variability of definitions for SSA that hamper the ability to draw a model for the risk factors and processes that facilitate it. Moreover, the role of suicide intent and planning in SSA is still unclear. Further studies should aim to clarify and refine the concepts and measures of SSA, thereby enabling more specific and concrete modeling of the psychological element in its formation.

**Conclusion:**

SSA is a distinguishable phenomenon that needs to be addressed specifically within the scope of suicidal behavior. Interpersonal problems, as well as impulsivity and aggression, seem to facilitate SSA when mental pain serves as a secondary factor. Healthcare professionals should be aware of SSA, and familiar with its specific risk factors. Moreover, psychological and suicidal risk assessment should include a designated evaluation of these risk factors as part of intervention and prevention models for SSA.

## Introduction

While the significance of identification, assessment, and intervention in the prevention of suicide attempts (SAs), and particularly, of suicide completion is undisputed, suicide completion is still recurrent at an annual yearly global age-standardized rate of 11.4 per one hundred thousand. Furthermore, approximately 10 times this rate attempt suicide annually (1). Thus, suicidologists are still faced with the challenge of identifying the factors leading to suicide (2, 3).

The first step toward identifying risk factors for suicide is to determine how to study this phenomenon. In general, suicidal behavior comprises a diverse set of behaviors, including suicidal thoughts, non-suicidal self-injuries, SAs, and actual death by suicide (4). Other related behaviors are deliberate self-harm, parasuicide, and non-suicidal self-injury (5). Given the significant differences between these behavioral categories, suicidologists are aware that the generalizability of research findings is dramatically affected by the population selected for study (4, 6). To date, one of the most prevalent methods for studying suicide is the psychological autopsy (7, 8). This strategy allows for very detailed information about the patient, including psychiatric records, police investigations, military records, etc. (9). However, while this approach can be informative as to certain suicide features, such as sociodemographic characteristics and psychiatric disorders, a serious limitation remains its lack of access to information pertaining to the subject’s difficulties, problems, and processes, particularly those of a more personal nature (10).

### Serious Suicide Attempt (SSA) Strategy

An alternative and promising strategy for investigating suicide focuses on the study of SSA survivors. As a concept, SSA is an SA which would have been fatal without the provision of speedy and effective first-aid care, other forms of emergency treatment or, in some cases, mere coincidence (11, 12).

Thus, SSA is an SA that requires hospitalization for more than 24 h and meets one of the following treatment criteria: (a) treatment in specialized units, including the intensive care unit; (b) surgery under general anesthesia; and (c) extensive medical treatment, including antidotes for drug overdose, telemetry, or repeated tests or investigations. In addition, attempted suicide by methods carrying a high-fatality risk (e.g., hanging or gunshot) is also defined as medically serious suicide attempt (MSSA) if the attempt led to hospitalization for over 24 h (13, 14). Individuals who have made SSAs may serve as valid proxies for studying suicides given that they are epidemiologically very much like those who complete suicide (13, 15), and twice as liable as other suicide attempters to subsequently complete suicide (16, 17).

Due to its emphasis on access to SSA survivors, this approach has several important advantages which have broadened the scope of potential research areas (14, 18, 19). Studying individuals who have survived a potentially lethal incident of self-harm enables a comprehensive inquiry into both the psychological processes leading up to the suicidal act, and the influence of early and current experiences that serve as catalysts for suicide (20, 21). Moreover, this approach may provide some insight into many first attempt suicide completers, who mental health professionals have so far neglected. Another advantage is the ability to follow patients who survived near-fatal attempts and explore their treatment outcomes (21, 22). To conclude, as Hawton postulated, “investigating survivors of SSAs can develop into a key research strategy in the study of suicidal behavior” [(20), p. 3].

Throughout the history of suicide research, authors have operationalized SSA’s definition in various ways, using widely differing criteria (e.g., hospitalization, suicide intent more than zero, violent method, etc.). This diversity of definitions, as well as of measures used and populations studied, make arriving at aggregate conclusions rather difficult.

Thus, the crucial question is how to best identify serious suicide attempters for studies of this kind. Drawing on Levi-Belz and Beautrais’ review (21) and several different studies in the field (13, 14, 18, 19), in this study we conceptualized SSA in terms of the attempt’s *actual medical lethality*. Medical lethality can be inferred from two different, but overlapping, dimensions: the suicidal act’s physiological consequences, and post-attempt medical procedures. Thus, we defined SSA as an SA that causes a significant physical injury requiring intensive, substantial medical treatment. Accordingly, we aimed to systematically review more recent studies whose definitions of SSA are based solely on these criteria. Our goal was to illuminate the psychological risk factors that relate to, or even facilitate, SSA, such as data regarding correlations, mediating roles, predictions, and comparisons between variables associated with SSA. As no such systematic review has been executed to date, we are hopeful that the results of this study will enhance knowledge of specific risk factors and psychological processes. This knowledge, in turn, may then facilitate SSA prediction, and enable researchers and clinicians to customize treatment programs for those at risk for SSA and suicide.

## Methods

### Information Databases and Searches

A comprehensive digital search strategy was applied to identify peer-reviewed articles on the relation between SSA and a wide range of psychological factors. This strategy is in line with Preferred Reporting Items for Systematic Reviews and Meta-Analyses (23). We also searched PubMed and Psycnet databases. Search terms were “serious” “OR” “near lethal” in combination with the Boolean “AND” operator with “suicide.” In addition, a manual search in Google Scholar was performed for hitherto unidentified studies.

### Study Selection

Studies were eligible if they were original, written in English, and presented data regarding a wide range of psychological factors associated with SSA. To focus solely on recent studies, we included studies from 2000. This time frame is also consistent with the emergence of the SSA strategy first proposed by Beautrais in studies from 1996 and 1999. Thus, we searched papers dating from January 1, 2000 to October 31, 2017. Case studies, reviews, book chapters, conference papers, and incomplete studies were excluded.

### Data Analysis

As studies on SSA risk factors are highly heterogeneous, with different study designs, measures, and sample types, they could not be combined into a singular meta-analysis study. Consequently, we conducted a systematic review of the results of each study. Studies were first categorized based on clusters of risk factors (e.g., mental pain, interpersonal factors, impulsivity, etc.), and then summarized while highlighting the information unique for each study, as well as features common to the studies in each cluster.

## Results

**Figure 1:**
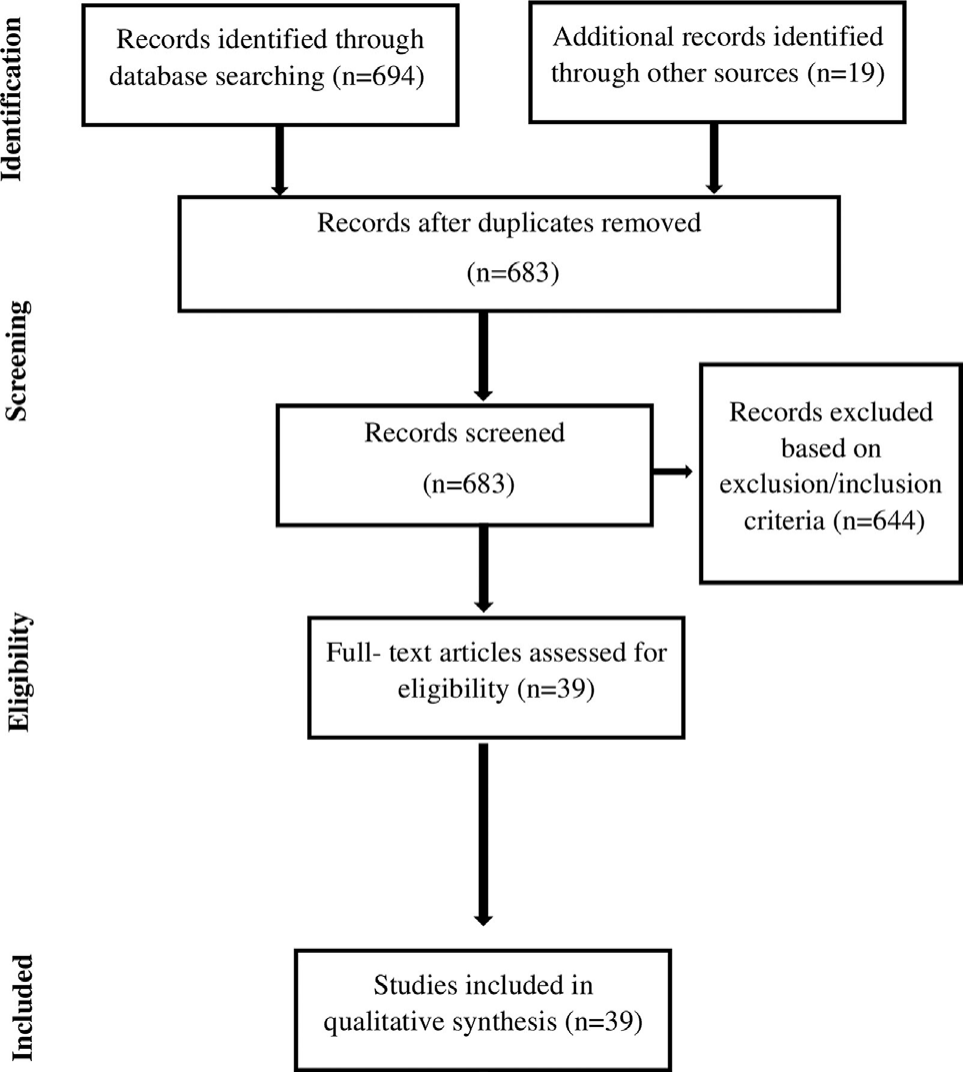
Dataset selection process.

The database search yielded a total of 683 citations. Based on both inclusion and exclusion criteria, a total of 39 original research studies were identified and included in the systematic review (see flowchart in Figure 1). In what follows, we present the literature review results, which have been sub-grouped according to common risk factor themes. A detailed description of reviewed studies is presented in Table 1.

**Table 1:** Characteristics of studies used in the review.

Number	Study	Title	Aim	Sample information
1.	Apter et al. (24)	Relationship between self-disclosure and serious suicidal behavior	To study the influence of self-disclosure on suicide attempters and completers	Sample size: 80 divided into four groups; G1 patients with suicidal thoughts but no suicidal behavior (20); G2 patients who made a mild SA (20); G3 patients who made an SSA (20); G4 patients who had no suicidal behavior (20)Mean age: G1 = 38.3 (SD = 15.0) years; G2 = 36.7 (SD = 17.2) years; G3 = 39.7 (SD = 18.9) years; G4 = 40.5 (SD = 16.1) yearsFemale: G1 = 55%; G2 = 55%; G3 = 55%; G4 = 50%

2.	Barnes et al. (25)	Help-seeking behavior prior to nearly lethal suicide attempts	To define various help-seeking behaviors prior to a nearly lethal suicide attempt in a population of adolescents and young adults and to examine the association between these behaviors and nearly lethal suicide attempts	Sample size: 666 divided into two groups; G1 hospitalized after an NLSA (153); G2 controls from population-based sample (513)Mean age: NAFemale: G1 = 46%; G2 = 57%

3.	Beautrais (13)	Suicides and serious suicide attempts: two populations or one?	To compare suicides and serious suicide attempts in terms of known risk factors for suicidal behavior	Sample size: 1,461 divided into three groups; G1 individuals who died by suicide (202); G2 individuals who made an MSSA (275); G3 control subjects (984)Mean age: G1 = 36.8 (SD = 16.2) years; G2 = 30.0 (SD = 14.2) years; G3 = NAFemale: G1 = 22.3%; G2 = 54.9%; G3 = NA

4.	Beautrais (26)	A case–control study of suicide and attempted suicide in older adults	To provide a descriptive profile of the sociodemographic, mental, and physical health characteristics and life circumstances of older people who make serious suicide attempts or die by suicide, and, using a case–control design, to compare these characteristics with those of a randomly selected community comparison series of adults of similar age	Sample size: 322 divided into three groups; G1 individuals who made an MSSA (22); G2 individuals who died by suicide (31); G3 comparison subjects (269)Mean age: G1 = 66.3 (SD = 9.2) years; G2 = 65.2 (SD = 8.2); G3 = 67.6 (SD = 8.5) yearsFemale: G1 = 68.2%; G2 = 35.5%; G3 = NA

5.	Beautrais (14)	Suicide and serious suicide attempts in youth: a multiple-group comparison study	To compare risk factors for suicide and medically serious non-fatal suicide attempts among youth under 25 years of age	Sample size: 336 divided into three groups; G1 individuals who committed suicide (60); G2 individuals who made an MSSA (125); G3 non-suicidal individuals (151)Mean age: G1 = 19.98 (SD = 2.53) years; G2 = 19.31 (SD = 3.1); G3 = 21.43 (SD = 1.52) yearsFemale: G1 = 18.3%; G2 = 54.4%; G3 = 48.3%

6.	Beautrais et al. (27)	Unemployment and serious suicide attempts	To examine the association between unemployment and risk of medically serious suicide attempt	Sample size: 1,330 divided into two groups; G1 individuals who made an MSSA (302); G2 non-suicidal individuals (1,028)Mean age: G1 = 30.4 (SD = 14.2) years; G2 NAFemale: G1 = 53.6%; G2 = NA

7.	Beautrais et al. (11)	Cannabis abuse and serious suicide attempts	To compare the relationship between cannabis abuse/dependence and risk of medically serious suicide attempts in individuals making serious suicide attempts and randomly selected comparison subjects	Sample size: 1,330 divided into two groups; G1 individuals who made an MSSA (302); G2 non-suicidal individuals (1,028)Mean age: NAFemale: NA

8.	Conner et al. (28)	Risk factors for suicide and medically serious suicide attempts among alcoholics: analyses of Canterbury Suicide Project data	To identify risk factors for serious suicidal behavior among individuals with alcohol dependence	Sample size: 146 participants with alcohol dependence divided into three groups; G1 completed suicide (38); G2 individuals who made an MSSA (62); G3 community controls (46)Mean age: NAFemale: NA

9.	Conner et al. (29)	Moderators of the relationship between alcohol dependence and suicide and medically serious suicide attempts: analyses of Canterbury Suicide Project data	To evaluate potential moderators of the relationship between alcohol dependence and suicide and medically serious suicide attempts by using case–control data gathered in the Canterbury region of New Zealand for the Canterbury Suicide Project	Sample size: 1,417 divided into three groups; G1 suicide decedents (193); G2 individuals who made an MSSA (240); G3 community controls (984)Mean age: G1 = 37.7 (SD = 15.9) years; G2 = 32.1 (SD = 14.0); G3 = 43.5 (SD = 17.7) yearsFemale: G1 = 22.8%; G2 = 52.5%; G3 = 51.6%

10.	Doihara et al. (30)	Trait aggression in suicide attempters: a pilot study	To investigate aggression in medically serious suicide attempters at an emergency department in Japan	Sample size: 126 divided into two groups; G1 hospitalized for an MSSA (55); G2 healthy control group (71)Mean age: G1 = 39.0 (SD = 16.6) years; G2 = 34.3 (SD = 11.3) yearsFemale: G1 = 60%; G2 = 71.8%

	Dombrovski et al. (31)	Lethal forethought: delayed reward discounting differentiates high- and low-lethality suicide attempts in old age	To examine the hypothesis that individuals who make less serious suicide attempts would show a preference for immediate rewards, whereas those who plan and carry out the most serious attempts would be more patient	Sample size: 80 divided into five groups; G1 individuals who made high-lethality SA (15); G2 individuals who made low-lethality SA (14); G3 “ideators” (12); G4 non-suicidal depressed (42); G5 psychiatrically healthy (31)Mean age: G1 = 67.4 (SD = 7.1) years; G2 = 66.1 (SD = 8.1) years; G3 = 69.5 (SD = 8.7) years; G4 = 70.3 (SD = 8.6) years; G5 = 68.1 (SD = 5.8)Female: G1 = 46.7%; G2 = 64.3%; G3 = 33.3%; G4 = 64.3%; G5 = 45.2%

11.	Donald et al. (32)	Risk and protective factors for medically serious suicide attempts: a comparison of hospital-based with population-based samples of young adults	To investigate risk and protective factors for medically serious suicide attempts among young Australian adults	Sample size: 475 divided into two groups; G1 hospitalized after an SA (95); G2 matched controls from population-based sample (380)Mean age: NAFemale: G1 = 48.4%; G2 = 48%

12.	Durant et al. (33)	Racial differences in hopelessness as a risk factor for a nearly lethal suicide attempt	To explore the relationship between hopelessness, race, and suicidal behavior	Sample size: 327 divided into two groups; G1 hospitalized after an NLSA (153); G2 controls from population-based sample (513)Mean age: NAFemale: G1 = 45%; G2 = 64%

13.	Elliott et al. (34)	A profile of medically serious suicide attempts	To identify factors associated with medically serious suicide attempts (requiring medical hospitalization)	Sample size: 97 divided into two groups; G1 individuals who made an MSSA (65); G2 individuals who made an MNSSA (32)Mean age: NAFemale: NA

14.	Fowler et al. (35)	Risk factors for medically serious suicide attempts: evidence for a psychodynamic formulation of suicidal crisis	To explore a psychodynamic model for suicide risk by examining risk factors for medically serious suicide attempts, including assessments of affect flooding, negative self-schema/fragmentation, and impaired reality testing, closely approximating Maltsberger’s psychodynamic formulation of suicide crisis	Sample Size: 75 divided into three groups; G1 inpatients with an MSSA (25); G2 inpatients with no suicidal activity (25); G3 outpatients with no suicidal activity (25)Mean age: G1 = 32.2 (SD = 11.3) years; G2 = 31.3 (SD = 14.7) years; G3 = 30.9 (SD = 14.4) yearsFemale: G1 = 76%; G2 = 68%; G3 = 68%

15.	Gvion et al. (19)	Aggression-impulsivity, mental pain, and communication difficulties in medically serious and medically non-serious suicide attempters	To evaluate the relative effects of aggression and impulsivity on the lethality of suicide attempts	Sample size: 196 divided into four groups; G1 hospitalized for MSSA (43); G2 MNSSA (49); G3 psychiatric control group (47); G4 healthy control group (57)Mean age: G1 = 37.37 (SD = 13.31) years; G2 = 40.31 (SD = 13.76) years; G3 = 40.96 (SD = 14.07) years; G4 = 37.28 (SD = 12.34) yearsFemale: G1 = 39.5%; G2 = 30.6%; G3 = 29.8%; G4 = 45.6%

16.	Gvion (36)	Aggression, impulsivity and their predictive value on medical lethality of suicide attempts: a follow-up study on hospitalized patients	To study the role of aggressive impulsive variants and suicide history in predicting the medical severity of follow-up suicide attempts	Sample size: 97 divided into three groups; G1 history of MSSA (33); G2 history of MNSSA (29); no history of suicide (35)Mean age: NAFemale: NA

17.	Horesh et al. (37)	Medically serious versus non-serious suicide attempts: relationships of lethality and intent to clinical and interpersonal characteristics	To investigate the relationship of intent and lethality in medically serious and medically non-serious suicide attempts and to examine relationship of specific psychological and clinical variables with the subjective and objective components of suicide intent	Sample size: 102 divided into two groups; G1 patients after an MSSA (35); G2 patients after an MNSSA (67)Mean age: G1 = 39.7 (SD = 15.3) years; G2 = 37.3 (SD = 14.0) yearsFemale: G1 = 48.6%; G2 = 53.7%

18.	Levi et al. (38)	Mental pain and its communication in medically serious suicide attempts: an “impossible situation”	To test the hypothesis that mental pain is a general risk factor for suicidal behavior and communication difficulties are a particular risk factor for medically serious suicidal behavior	Sample size: 173 divided into three groups; G1 hospitalized for an MSSA (35); G2 MNSSA (67); G3 without psychiatric diagnosis or history of suicidal behavior (71)Mean age: G1 = 39.7 (SD = 15.3) years; G2 = 37.3 (SD = 14.0) years; G3 = 36.5 (SD = 14.0) yearsFemale: G1 = 48.6%; G2 = 53.7%; G3 = 47.9%

19.	Levi-Belz et al. (39)	Attachment patterns in medically serious suicide attempts: the mediating role of self-disclosure and loneliness	To examine the associations between attachment patterns to severe suicidal behavior	Sample size: 102 divided into two groups; G1 patients after an MSSA (35); G2 patients after an MNSSA (67)Mean age: G1 = 39.7 (SD = 15.3) years; G2 = 37.3 (SD = 14.0) yearsFemale: G1 = 48.6%; G2 = 53.7%

20.	Levi-Belz et al. (18)	Mental pain, communication difficulties, and medically serious suicide attempts: a case–control study	To assess the role of mental pain and communication difficulties in MSSA	Sample size: 336 divided into four groups; G1 hospitalized for MSSA (78); G2 MNSSA (116); G3 psychiatric control group (47); G4 healthy control group (95)Mean age: G1 = 38.5 (SD = 14.2) years; G2 = 38.5 (SD = 13.9) years; G3 = 40.9 (SD = 14.0) years; G4 = 38.5 (SD = 14.2) yearsFemale: G1 = 43.6%; G2 = 56.0%; G3 = 70.2%; G4 = 45.3%

21.	Lohner and Konrad (40)	Deliberate self-harm and suicide attempt in custody: distinguishing features in male inmates’ self-injurious behavior	To find differences between self-injurious behavior of “low seriousness” (i.e., low-lethality and low-suicidal intent) and of “high seriousness,” by inmates while under custodial authority	Sample size: 49 male inmates exhibiting self-injurious behavior and high-lethal suicide attemptsMean age: 27.1 (SD = 9.152) years

22.	Lopez-Castroman et al. (41)	Post-traumatic stress disorder following childhood abuse increases the severity of suicide attempts	To investigate the association of PTSD and childhood abuse to suicide attempts	Sample size: 726 suicide attemptersMean age: NA (median)Female: 74.4%

23.	Lopez-Castroman et al. (42)	Heavy tobacco dependence in suicide attempters making recurrent and medically serious attempts	To investigate, specifically, the association between the level of tobacco dependence and the severity of suicidal outcomes among suicide attempters, as well as the relationship of impulsivity with both conditions	Sample size: 542 hospitalized patients in a unit for affective disorders and suicidal behaviorMean age: NA (median—41.1)Female: 73.6%

24.	Marzano et al. (43)	Psychiatric disorders in women prisoners who have engaged in near-lethal self-harm: case–control study	To investigate prevalence of psychiatric disorders in women prisoners who had recently engaged in near-lethal self-harm (cases) and others who had never carried out near-lethal attempts in prison (controls)	Sample size: 120 female prisoners divided into two groups; G1 made an NLSA (60); G2 matched controls (60)Mean age: NA (median: G1-25.5, G2-26)

25.	Marzano et al. (44)	Psychosocial influences on prisoner suicide: a case–control study of near-lethal self-harm in women prisoners	To examine the psychosocial influences on female prisoner suicide by carrying out a study of near-lethal self-harm	Sample size: 120 female prisoners divided into two groups; G1 made an NLSA (60); G2 matched controls (60)Mean age: NA (median)

26.	McGirr et al. (45)	Deterministic learning and attempted suicide among older depressed individuals: cognitive assessment using the Wisconsin Card Sorting Task	To characterize the relationship between suicidal behavior and cognitive control during learning in a complex environment among older individuals	Sample size: 93 divided into four groups; G1 individuals who made high-lethality SA (14); G2 individuals who made low-lethality SA (20); G3 non-suicidal depressed (29); G4 healthy controls (30)Mean age: G1 = 68.86 (SD = 7.53) years; G2 = 66.80 (SD = 8.15) years; G3 = 70.30 (SD = 9.03) years; G4 = 69.77 (SD = 6.76) yearsFemale: G1 = 50%; G2 = 50%; G3 = 65.5%; G4 = 46.7%

27.	Potter et al. (46)	The influence of geographic mobility on nearly lethal suicide attempts	To understand the relationship between mobility and suicidal behavior by studying the association at the individual level of analysis, in a large sample, and using a rigorous measure of suicidal behavior	Sample size: 666 divided into two groups; G1 cases of NLSA (153); G2 control subjects (513)Mean age: NA (range 13–34)Female: G1 = 45.8%; G2 = 56.9%

28.	Powell et al. (47)	Alcohol consumption and nearly lethal suicide attempts	To examine various pathways that may link alcohol consumption and suicide, which might be used to identify persons at higher risk of suicide	Sample size: 666 divided into two groups; G1 hospitalized after an NLSA (153); G2 controls from population-based sample (513)Mean age: NAFemale: G1 = 45.8%; G2 = 56.9%

29.	Rivlin et al. (48)	Psychiatric disorders in male prisoners who made near-lethal suicide attempts: case–control study	To investigate the association of psychiatric disorders with near-lethal suicide attempts in male prisoners	Sample size: 120 male prisoners divided into two groups; G1 made an NLSA (60); G2 matched controls (60)Mean age: NA

30.	Rivlin et al. (49)	The suicidal process in male prisoners making near-lethal suicide attempts	To identify the psychological problems and processes leading up to, and following, suicide attempts in order to identify key opportunities for prevention	Sample size: 60 male prisoners who had made NLSAMean age: NA (median—29 years)

31.	Simon et al. (50)	Characteristics of impulsive suicide attempts and attempters. Suicide and life-threatening behavior	To test four hypotheses concerning the characteristics of individuals who make impulsive and non-impulsive suicide attempts	Sample size: 666 divided into two groups; G1 cases of NLSA (153); G2 control subjects (513)Mean age: NA (range 13–34)Female: NA

32.	Soloff et al. (51)	High-lethality status in patients with borderline personality disorder	To identify risk factors for suicide within a BPD sample by comparing patients with high-and low-lethality attempts	Sample size: 113 borderline personality disorder attempters divided into two groups; G1 high-lethality attempters (44); G2 low-lethality attempters (69)Mean age: G1 = 31.3 (SD = 8.8) years; G2 = 27.5 (SD = 7.6) yearsFemale: G1 = 63.6%; G2 = 76.8%

33.	Swahn and Potter (52)	Factors associated with the medical severity of suicide attempts in youths and young adults	To determine how demographic factors, symptoms of mental health problems, help-seeking behaviors, and the characteristics of the suicide attempts are associated with the severity of outcomes from non-fatal suicide attempts	Sample size: 200 suicide attempters divided into two groups; G1 with NLSA (153); G2 with LLSA (47)Mean age: NA (range 13–34)Female: G1 = 45.8%; G2 = 61.7%

34.	Swann et al. (53)	Increased impulsivity associated with severity of suicide attempt history in patients with bipolar disorder	To investigate the relationship between impulsivity and severity of past suicidal behavior, a potential predictor of eventual suicide, in patients with bipolar disorder	Sample size: 48 subjects with bipolar personality disorder divided into three groups; G1 history of an MSSA (8); G2 history of an MNSSA (16); G3 without history of SAMean age: G1 = 34.3 (SD = 5.5) years; G2 = 35.1 (SD = 8.3) years; G3 = 35.0 (SD = 12.1) yearsFemale: G1 = 62.5%; G2 = 68.8%; G3 = 37.5%

35.	Szanto et al. (54)	The cost of social punishment and high-lethality suicide attempts in the second half of life	To understand the role of social decision making in suicide, our study focused on older adults because of the high proportion of medically serious suicide attempts in this age group	Sample size: 103 divided into four groups; G1 individuals who made high-lethality SA (26); G2 individuals who made low-lethality SA (20); G3 non-suicidal depressed (35); G4 non-psychiatric controls (22)Mean age: G1 = 62.8 (SD = 10.1) years; G2 = 62.5 (SD = 6.4) years; G3 = 66.9 (SD = 7.2) years; G4 = 64.6 (SD = 11.0) yearsFemale: G1 = 35%; G2 = 40%; G3 = 60%; G4 = 53%

36.	Szanto et al. (55)	Decision-making competence and attempted suicide	To examine the susceptibility of low-lethality and high-lethality suicide attempters to common decision biases, which may ultimately obscure alternative solutions and deterrents to suicide in a crisis	Sample size: 171 divided into five groups; G1 individuals who made high- lethality SA (31); G2 individuals who made low-lethality SA (29); G3 “ideators” (30); G4 non-suicidal depressed (53); G5 psychiatrically healthy (28)Mean age: G1 = 64.0 (SD = 9.6) years; G2 = 62.0 (SD = 7.4) years; G3 = 65.1 (SD = 10.7) years; G4 = 69.4 (SD = 8.7) years; G5 = 68.4 (SD = 12.0)Female: G1 = 48%; G2 = 48%; G3 = 40%; G4 = 55%; G5 = 57%

37.	Trakhtenbrot et al. (22)	Predictive value of psychological characteristics and suicide history on medical lethality of suicide attempts: a follow-up study of hospitalized patients	To examine the role of mental pain, communication difficulties, and suicide history in predicting the medical severity of follow-up suicide attempts	Sample size: 153 divided into three groups; G1 hospitalized for an MSSA (53); G2 hospitalized for an MNSSA (64); G3 inpatients without a history of suicide (36)Mean age: G1 = 37.60 (SD = 12.25) years; G2 = 37.74 (SD = 13.05) years; G3 = 40.27 (SD = 13.26) yearsFemale: G1 = 42%; G2 = 39%; G3 = 31%

38.	Vanyukov et al. (56)	Perceived burdensomeness is associated with low-lethality suicide attempts, dysfunctional interpersonal style, and younger rather than older age	To answer the questions: does a high level of perceived burdensomeness differentiate medically serious suicidal acts, most closely resembling death by suicide, from less serious ones? How is perceived burdensomeness related to dysfunctional personality dimensions implicated in suicide?	Sample size: 165 aged over 42 divided into five groups; G1 individuals who made high-lethality SA (32); G2 individuals who made low-lethality SA (32); G3 suicidal ideators (34); G4 non-suicidal depressed individuals (37); G5 non-psychiatric controls (30)Mean age: G1 = 65.50 (SD = 11.0) years; G2 = 61.25 (SD = 7.1) years; G3 = 64.47 (SD = 10.1) years; G4 = 66.68 (SD = 5.9) years; G5 = 67.57 (SD = 11.7)Female: G1 = 44%; G2 = 50%; G3 = 38%; G4 = 38%; G5 = 60%

39.	Wiktorsson et al. (57)	Medically serious and non-serious suicide attempts in persons aged 70 and above	To compare clinical and psychosocial characteristics in older adult attempters (70+) with and without medically serious suicide attempts	Sample size: 101 older adult suicide attempters divided into two groups; G1 with MSSA (28); G2 without MSSA (73)Mean age: G1 = 79.5 years; G2 = 79.8 yearsFemale: G1 = 60.7%; G2 = 50.7%

### Psychopathology

Given its strong linkages with suicide (58–60), psychopathology was most likely to constitute a risk factor for SSAs. Several studies have examined the relationships between SSA and specific DSM mental disorders (depressive disorder, borderline personality disorder, etc.). Led by Beautrais, The Canterbury Suicide Project in New Zealand was one of the pioneer studies in the field of SSAs (13, 14). Beautrais (14) examined 125 young (under 25) individuals who made SSAs, and compared them to 151 healthy controls. Also in the framework of this project, Beautrais et al. (61) found that those who executed SSAs had high levels of mental disorders (according to the DSM-III-R diagnoses) and psychiatric comorbidity at the time of the SA. More specifically, they found that the probability that SSA attempters were diagnosed with major depressive disorder (MDD), anxiety disorders, and anti-social behaviors was higher when compared with non-suicidal subjects (14). In another study, Beautrais (26) found similar results: current mood disorders elevated the risk for SSAs mostly among older adults [odd ratio (OR) = 179]. Psychiatric hospital admission within the previous year was also found to elevate the risk for SSAs [OR = 24.4 (26)]. Interestingly, the strong relationship between depression and suicidal behavior, and SSAs was even found on the temperamental level, whereas Rihmer et al. (62) found that four of the five affective temperaments contained depressive components (i.e., depressive, cyclothymic, irritable, and anxious).

Rivlin et al. (48) focused on male prisoners who made SSAs, and compared them to 60 prisoners who had never performed near-lethal SAs in prison (control group). The results showed that psychiatric disorders and specific mood disorders are highly common among serious suicide attempters. Specifically, psychiatric disorders were present in all SSA cases, while present in only 62% of the controls. The current psychiatric disorders most associated with SSAs included major depression (OR = 42.0), psychosis (OR = 15.0), and anxiety disorders (OR = 6.0). Marzano et al. (43) employed the same design on 60 female prisoners and on controls. Here too, the strongest associations were between SSAs and current depression (OR = 23.7), and with the history of psychiatric in-patients’ admissions (OR = 25.4). Other studies reached similar results (32, 35). Lopez-Castroman et al. (41) investigated 726 adult patients who had attempted suicide, of which 104 met SSA criteria. The authors found that individuals who made SSAs had a significantly higher probability of receiving a diagnosis of PTSD (OR = 1.86). Interestingly, when combined with a lifetime diagnosis of PTSD, individuals who experienced different types of childhood abuse (emotional, physical, and/or sexual) showed an increased risk for serious attempts (up to OR of 4.14).

Another factor for SSA is substance abuse disorders, including alcohol, cannabis, cocaine, and opiate abuse/dependence. The research clearly shows that substance abuse is strongly correlated with suicidal behavior. Thus, cannabis use/dependence is commonly attributed to suicide attempters or completers who reported some additional risk factors for suicide, such as mood disorders, stressful life events, interpersonal problems, poor social support, loneliness, and feelings of hopelessness (63).

Although there are numerous studies on the linkage between suicide behavior and substance use disorder (SUD), the literature regarding the association between SUD and SSA has been limited since Beautrais, Joyce, and Mulder’s pioneering study in 1999 (11). In this study, 302 instances of SSA were compared with 1,028 healthy controls from the local community. 16.2% of those who performed SSAs met DSM criteria for cannabis abuse/dependence at the time of the SA, while among the control group, only 1.9% met DSM criteria for cannabis abuse/dependence.

In another study, conducted in the framework of the Canterbury Suicide Project, Conner et al. (29) used case–control data to assess potential moderators for the connection between alcohol dependence and suicide or MSSAs. The psychological autopsy methodology was used to collect data on 193 suicide decedents, 240 individuals who engaged in MSSAs, and 984 adult community controls. The correlation between alcohol dependence and suicide (excluding high-lethal attempts) was intensified with increased age. Neither mood disorder nor gender moderated the relationship between alcohol dependence and suicide. Increased age strengthened the association between mood disorder and suicide, while decreased age amplified the association between mood disorder and near-lethal attempts.

Another study in the same project (28) looked for risk factors for serious suicidal behavior among individuals with alcohol dependence. The cohort included 38 completed suicides, 62 individuals whose SSAs had medical repercussions, and 46 community controls who had experienced alcohol dependence in the past month. Mood disorders and financial problems were more frequent among MSSA attempters compared with the controls. Individuals who had completed suicides, mostly older males, were more prone to mood disorders, partner/relationship difficulties, and other interpersonal life events than the controls.

Powell et al. (47) assessed the linkage between SSAs, lethal SAs, and aspects of alcohol consumption (drinking frequency and quantity, binge drinking, alcoholism, drinking within 3 h prior to the SA, and the age one began drinking) among patients aged 13–34. All measures were found to be related to near-lethal SAs. Odds ratios ranged from 2.4 for alcoholism to 7.0 for drinking within 3 h prior to the attempt. All exposure variables, except the age one started drinking, exhibited a J-shaped relationship between alcohol exposure and near-lethal SAs. Once controlling for potential confounders and other measures of alcohol exposure, drinking within 3 h prior to the attempt remained most strongly associated (ORs > 6) with the seriousness of the attempt.

Lopez-Castroman et al. (42) aimed to investigate the specific association between the tobacco dependence level and the severity of suicidal outcomes. A cohort of 542 adult suicide attempters was assessed, of them 107 who made SSAs. SSA attempters were characterized as smokers, compared with non-smokers. The authors concluded that high or very high levels of tobacco dependence could point to a specific vulnerability leading to more severe SAs.

To conclude, the paucity of research regarding the relation between substance abuse/dependence and SSA calls attention to the need for further research to understand the impact of substance abuse/dependence on SSA.

### Mental Pain Levels: Experiences of Depression, Anxiety, Hopelessness, and Distress

As Shneidman postulated in his seminal book, “*Suicide is caused by psychache*” (Shneidman, 1993, p. 51), mental pain is one of the main facilitators of SAs in general, and particularly in suicide. Hence, the proliferation of literature on the relationships between the subjective experience of mental pain, the phenomenological experience of depression, anxiety, and hopelessness, and SSAs.

Several studies have shown that SSAs are characterized by high levels of mental pain, specifically hopelessness, when compared with healthy controls. Examining several personality traits and psychological states, Beautrais et al. (11) demonstrated that hopelessness is a main risk factor for SSA, as well as neuroticism and low-self-esteem. Other studies arrived at similar results. Durant et al. (33), for example, conducted a case–control study of SSAs in the United States to reveal racial differences. They compared 105 SSA attempters with 395 controls selected through a random-digit-dial telephone survey. Compared with the controls, the SSA attempters, especially young African Americans, reported significantly higher levels of hopelessness.

While these studies demonstrated that hopelessness levels are significantly higher among SSA attempters than in the general population, results were different when SSA subjects were compared with a more suitable comparison group, such as hospitalized patients or individuals who made SAs that were not considered serious. When SSA patients were compared with patients with no history of suicidality, different result patterns pertaining to psychopathology levels emerged. In the Center for Disease Control and Prevention Project in Houston, Swahn and Potter (52) compared 153 nearly lethal attempters with 47 less lethal attempters on several demographic and psychological variables. They found that the percentage of subjects who experienced severe depression (not a DSM definition) in both groups was significantly high—86% of SSA attempters and 98% of those who made less lethal attempts. Moreover, depression was statistically associated with a lower risk for SSAs.

Like the Houston project, the Israeli SSAs project investigated 78 SSA attempters and compared them with 116 suicide attempters whose attempts were less serious, 47 psychiatric controls with no past accounts of suicidal behavior, and 95 healthy controls (18). While depression experience levels measured by the Beck Depression Inventory were considerably higher in the SSA group than in the psychiatric or healthy control group, no differences related to depression were found when comparing individuals who executed SSAs with those whose attempts were less medically serious. A similar tendency was found when overlapping aspects of distress—mental pain, anxiety, and hopelessness—were examined [e.g., Ref. (19)]. For example, while hopelessness was identified as a predictor of SSAs when compared with controls, it was unable to predict medical lethality when SSA attempters were compared with non-SSA attempters [(38), see also (64)]. Mental pain experience, measured by the OMMP scale (65), yielded the same result pattern.

Wiktorsson et al. (57) comparison between older adult attempters with (*n* = 28) and without (*n* = 73) SSAs, produced comparable results. They found that while major depression was common in both groups, SSA attempters were higher on the anxiety scale. When asked about the reasons for attempting suicide, SAA attempters ascribed the attempt to interpersonal social problems and functioning difficulties more often than the non-suicidal group.

Vanyukov et al. (56) examined the perceived burdensomeness factor associated with the interpersonal theory of suicide (66) alongside other psychological factors (e.g., Big Five factors, impulsivity, and anger rumination). They compared levels of perceived burdensomeness among depressed suicidal subjects with a history of both high- and low-lethality attempts, depressed subjects with suicidal ideation, non-suicidal depressed subjects, and psychiatrically healthy controls. Surprisingly, perceived burdensomeness was higher among low-lethality suicide attempters compared with all the other groups, including SSA attempters. Older adults SSA attempters scored very low on perceived burdensomeness, implying that, contrary to thwarted belongingness, the perceived burdensomeness factor does not figure significantly in the SA’s lethality.

Lohner and Konrad (40) examined several risk factors among inmates who were high- and low-lethality suicide attempters while under custodial authority. The seriousness of the attempt was positively correlated with measures of depression, and negatively correlated with the psychopathy factor, measured by the Psychopathy Checklist—revised (67). No other psychopathological or mental pain factors were found to be related to seriousness of the attempt (hopelessness, cluster B personality disorder, etc.).

Regarding suicide intent, Horesh et al. (37) showed that high levels of mental pain was a significant factor in the prediction of the subjective components of the suicide intent scale which measures the intent to die. However, mental pain did not predict the objective components of suicide intent, thereby bearing a significant association with lethality of the suicidal attempt.

Together, these results highlight that while distress and mental pain levels are high among suicide attempters, they do not characterize only SSA attempters, as non-SSA attempters also report high-distress levels (which may lead them to attempt suicide in the first place). In other words, while psychopathology, as well as mental pain variables (e.g., hopelessness), are important risk factors for SAs in comparison with the general population, they are not indicative of differences between SAs’ low and high levels of seriousness. Although, as Shneidman (68) argued, psychological pain is the common stimulus for both suicide and suicidal attempts, it is only when high levels of mental pain are accompanied by other psychological dimensions, such as interpersonal difficulties, impulsivity, or decision-making deficits, that they can increase the risk for SSAs. Found in several studies, these interactions (19, 38) will be elaborated on later in this review.

### Interpersonal Factors

Several studies examined the contribution of different factors to SSAs within the interpersonal context. First and foremost, interpersonal and communication difficulties were found to be strongly related to SSAs. Levi et al. (38) investigated 35 SSA attempters and compared them with two control groups: suicide attempters whose attempts were less serious, and healthy controls. They found that self-disclosure and perceived loneliness are unique predictors of SSAs, above and beyond mental pain levels (i.e., depression and hopelessness). Specifically, levels of self-disclosure, represented by difficulties in sharing intimate information with significant others, was considerably lower among SSA attempters in comparison with the other two control groups. Moreover, self-disclosure was the sole predictor that contributed both to the attempt’s medical lethality, and to the type and extent of the medical treatment received after the attempt. Perceived loneliness was highly predictive of the attempt’s severity. Thus, the authors proposed a model which they coined “an impossible situation” to describe instances that involve individuals who are unable to ask for help (hence “impossible”). In these cases, the matching of the predisposition for mental pain with other factors can provoke an SA with serious medical ramifications or an actual suicide (38). Apter et al. (24) arrived at similar results in their investigation of depressed patients. In particular, they found that self-disclosure was significantly lower among SSA attempters than among non-SAA attempters, suicide ideators, and patients without a history of suicide.

In a more recent study, Levi-Belz et al. (18) demonstrated that the communication difficulties dimension impacted both the distinction between MSSA attempters and non-MSSA attempters, and from psychiatric controls. Interestingly, the interaction between mental pain and communication difficulties accounted for 23% of the suicide lethality variance, above and beyond each component’s individual contribution. These findings demonstrated that the severity of the attempt depends on the individual’s ability to communicate their distress to others. Schizoid tendency and loneliness were the most valid sole predictors within the communication difficulties dimension. Gvion et al. (19) confirmed the results pertaining to the interaction between mental pain and schizoid tendency in a larger sample of MSSA and non-MSSA attempters.

In another study from the Israeli project, Horesh et al. (37) showed that difficulties in self-disclosure contributed significantly to the prediction of the objective components of the suicide intent scale, which in turn, determined the attempt’s medical lethality. Thus, low-interpersonal communication abilities facilitated higher medical lethality and higher intent that were reflected in objective circumstances related to the attempt (including preparation and steps taken to avoid discovery). In line with these results, it was found that among suicide attempters over 70-years-old, SSA attempters were more likely to attribute their attempts to social problems than non-SSA attempters (57).

When examining the core personality factors that may facilitate communication difficulties, Levi-Belz et al. (39) showed that attachment style significantly predicted the severity of the suicidal attempt, above and beyond the contribution of mental pain. Specifically, the more avoidant the subject’s attachment style was, the higher the level of their attempt’s medical lethality was. The authors also confirmed a structured equation model in which avoidant attachment facilitated medical lethality through the mediation of social support and self-disclosure. Interestingly, Signoretta et al. (69). found that subjects with communication difficulties and low-social interaction were mostly characterized by depressive temperament traits. This may highlight the interchange relationship between mental pain features and interpersonal factors which are main factors in our model for SSAs.

Other studies explored more directly the ability of SSA attempters to ask for help. Beautrais (14) employed a series of personality and cognitive style measures to assess SSA attempters (*n* = 125) and non-suicidal community comparison subjects (*n* = 151). SSA attempters were characterized by significantly lower levels of actual social interaction. When dichotomized, those with low levels of social interaction had elevated odds (up to eight times more) for SSA than controls.

Barnes et al. (25) study focused solely on subjects seeking help from others. They found that 153 subjects aged 13–34 who made SSAs had been less likely than the random sample of 513 control subjects to seek help from any consultant, including psychologists and physicians, in the past month.

To conclude, difficulties in interpersonal communication—reflected by a low ability to disclose oneself, resulting in loneliness and low-social support—seem to play an important role in more lethal SAs associated with high-objective suicide intent. At the basis of these difficulties are characteristics of avoidant attachment style, as well as schizoid tendency. Most of the studies indicated that interpersonal difficulties become risk factors when there is a high level of distress, depression, and mental pain in the background, thereby pointing to the interaction between mental pain and interpersonal problems as an important risk factor for SSAs.

### Decision-Making Factors

Over the last five decades, the fields of psychiatry and psychology have been influenced by economic theories that study patterns of decision making. Decision-making processes result in choosing a course of action or perception from several alternatives. To make optimal choices, our brain screens, gathers, and analyzes information prior to each decision. Recently, studies have begun to look at the impulsivity factor of suicidal behavior as a construct that involves a failure of higher-order control, that is, a deficit in decision making (19, 31). Thus, poor decision making can ultimately obscure alternative solutions and lead to suicidal behavior in times of crisis or depression.

The association between decision making and attempt lethality is mostly studied among middle-aged and elderly subjects who traditionally have high rates of both significantly lethal attempts and suicide (55). In Dombrovski et al. (31) seminal study, four groups of depressed participants aged 60 and older were assessed: 15 high-lethality suicide (SAA) attempters, 14 low-lethality suicide attempters, 12 who contemplated suicide, and 42 depressed subjects with no history of suicidal thoughts. The reference group comprised of 31 psychiatrically fit elders. To measure the preference for smaller immediate versus larger delayed rewards, participants were tested on Kirby’s Monetary Choice Questionnaire. Interestingly, the SSA attempters were more disposed to delay future rewards than the low-lethality attempters. Moreover, low-lethality attempters exhibited an exaggerated preference for immediate rewards compared with the two control groups. These effects were stable also after accounting for several possible covariates, such as education, global cognitive function medications, and possible brain injury. In another study, McGirr et al. (45) assessed older suicide attempters of varying medical lethality. All participants completed the Wisconsin Card Sorting Test that examines cognitive control during rule learning. Cognitive control is a mental process that underlies rule learning by integrating feedback with past knowledge of contingency and environment structure. In contrast to low-lethality attempters and healthy controls, high-lethality attempters exhibited poorer conceptual reasoning and increased rates of perseverative errors and total errors. Moreover, high-lethality attempters made more conceptual errors than non-suicidal depressed participants. Thus, this study highlights that high-lethality SAs in older people are related to impaired cognitive control during rule learning. This liability may contribute to serious or even fatal suicidal acts in old age.

To better define and map the diversity in SA lethality in terms of specific decision-making deficits, Szanto et al. (55) evaluated 31 older and middle-aged SSA attempters, and compared them, on decision biases, with 29 low-lethality suicide attempters, 30 suicide ideators, 53 non-suicidal depressed subjects, and 28 psychiatrically well participants. Attempters, ideators, and non-suicidal depressed subjects had unipolar non-psychotic major depression. The authors found that all attempters (both SSAs and those with lower lethality) were more susceptible to *framing effects* (i.e., made decisions affected by irrelevant variations in how information is presented) in comparison with the control groups. Thus, it seems that SSA attempters and low-lethality attempters are similar in their tendency to make decisions influenced by irrelevant variations in the way information is presented to them. This may indicate that suicidal behavior, but not its severity, reflects a lack of ability to make a decision from an objective standpoint during a crisis.

In another study, Szanto et al. (54) used the *Ultimatum Game* in which players decide whether or not to accept dubious monetary offerings from another player. Participants were older adults suffering from depression with a past of high-lethality SAs, low-lethality SAs, and no suicidal attempts. The control group consisted of healthy controls. Participants in all groups penalized their counterparts in response to inequitable monetary offers. Yet, low-lethality attempters, non-suicidal depressed participants, and healthy controls decreased the extent of penalty they inflicted as the level of punishment inflicted upon them increased—they accepted more unfair offers as the risks grew higher. SSA attempters did not modify their choices in accordance with stake magnitude, and penalized unjust offers regardless of the cost. 66% of the difference between the low-lethality attempters and non-psychiatric controls was explained in terms of individual differences in fairness judgments: the comparison group judged offer fairness as a combined function of inequality and magnitude, whereas the SSA attempters judged offer fairness based on inequality.

To conclude, decision-making impairments are risk factors for suicide behaviors in general. Nevertheless, specific difficulties, i.e., the lack of ability to calculate the positive and negative consequences of one’s actions, and the impaired ability to modify choices when making decisions regarding actions, are related to more serious suicide behaviors. As most research on decision making and SSAs focuses on the elderly population, generalization of conclusions is limited.

### Impulsivity–Aggression Factors

Under the concept of aggression, the literature employs diverse terms, such as violence, aggression, irritability, and hostility. These constructs have common aspects and are highly correlated (5, 70). Thus, we will relate to these constructs interchangeably. Several studies have highlighted the importance of aggression and related concepts to SSAs. Doihara et al. (30) studied the aggression dimension in 55 MSSA attempters in a hospital’s emergency room, and compared them with a control group of 71 healthy individuals. It was found that aggression and hostility scores were significantly higher in the SSA attempters when compared with controls.

The study of aggression is usually associated with the study of impulsivity as some researchers suggest that the overlap between these constructs is strong, and therefore they should be viewed as a single phenotype [for review see Ref. (5)]. Accordingly, Soloff et al. (51) evaluated 44 high-lethality attempters and 69 low-lethality attempters. Impulsivity, aggression, and state hostility failed to distinguish between high- and low-lethality attempters. Gvion et al. (19) studied 43 MSSA attempters, 49 medically non-SSA attempters, 47 psychiatric patients who had not attempted suicide, and 57 healthy control subjects. Among other variables, participants were assessed for aggression–impulsivity. The data revealed that although aggressive variables (e.g., anger-in, anger-out, and violence) and impulsivity differentiated between suicide attempters and non-attempters, they did not differentiate between high- and low-lethality attempters.

Interestingly, in a recent follow-up study (2017), Gvion examined the role of aggressive-impulsive variants and suicide history in foreseeing the medical severity of follow-up SAs. Anger-out, violence, and impulsivity were significantly and positively correlated with medical severity of follow-up SAs. Impulsive–aggressive variables accounted for 10.6% of the variance, over and above the contribution of personal factors, medical severity of index SA, and hopelessness. Moreover, interactions between medical acuteness levels of the index SA and impulsivity, self-disclosure, and anger-in, accounted for 16.4% of the variance, over and beyond each component alone. The findings further indicate that although impulsivity and aggression do not necessarily account for the seriousness of the original attempt (19), they do facilitate the ability to attempt a more lethal suicide in the future. This is especially true among those who originally made an SSA.

An interesting distinction in the literature relates to state or trait characteristic of impulsivity (71). A common way to operationalize state impulsivity is to examine the degree of objective signs of planning. Rivlin et al. (49) investigated 60 prisoners who performed near-lethal SAs. Although 73% indicated that they had intended to die, 40% of the acts were impulsive. The prisoners said they had thought about and planned the act for only a very short period of time (typically less than 3 h). Moreover, very few prisoners took any precautions against detection, left a suicide note, made plans or arrangements for suicide, or thought about the timing of the act.

Simon et al. (50) studied 153 subjects aged 13–34 who made nearly lethal suicide attempts. 24% of survivors contemplated their attempt for under 5 min. Those who executed their attempt within 5 min of the decision were less likely to have considered another method of suicide.

Swann et al. (53) compared bipolar SSA attempters, non-SSA attempters, and bipolar non-attempters. Subjects were assessed using the Barratt Impulsiveness Scale (BIS) and behavioral laboratory performance measures (immediate memory/delayed memory tasks). While no difference was found between the groups on BIS scores, impulsivity as manifested in behavioral laboratory performance measures was greatest in those with the most serious medical attempts. These results can also be explained in terms of the difference between trait and state aspects of impulsivity.

To summarize, aggressive–impulsive variables play a role in suicide behavior in general. Regarding SSA, data are sparse and limited. Looking at aggression and impulsivity in SSAs may differ significantly from studying aggression and impulsivity among SA repeaters and/or those who make low-lethality attempts. The extent of preparation and planning is a significant factor in determining the seriousness of the attempt, and is generally used as an indicator of state impulsivity. Nonetheless, many people die by suicide or make high-lethal acts performed on the spur of the moment with no or limited planning. The lethality of these attempts depends also on circumstantial factors (like availability of means). The findings further indicate that although impulsivity and aggression do not necessarily account for the lethality of the original attempt, they are factors in the ability to attempt a more lethal suicide in the future. This is especially true among those who originally made an SSA.

### Other Risk Factors for SSA

In this section, our focus is on specific and more isolated factors, rather than on risk factor clusters. Considering these factors’ scarcity, we have also included studies published 5 years prior to 2000. It goes without saying that more research is needed to fully appreciate the accuracy and specific impact of these variables as risk factors for SSA.

Negative life events were one of the factors suggested to be an isolated risk factor for SSAs. In the Canterbury Suicide Project, Beautrais (14) found a higher number of stressful life events among young people who made SSAs compared with controls with no history of suicidal behavior. Specifically, childhood and family adversities (e.g., sexual abuse or poor parenting) were significantly higher among the SSA group. Similar results were found in Elliott et al. (34) study that examined demographic and psychiatric factors associated with serious suicide attempters when compared with 32 non-medically serious attempters admitted to emergency rooms. Serious suicide attempters were found to have a higher rate of sexual and physical abuse, and in general, higher numbers of traumatic life events. They were also found to have higher rates of borderline personality disorder.

Other studies that investigated the relationship between medical lethality and life events among specific groups had similar results. Marzano et al. (44) studied 60 imprisoned male serious suicide attempters and compared them with inmates who had not attempted suicide. The Childhood Trauma Questionnaire was used to assess accounts of past emotional, physical, and sexual abuse, and emotional and physical neglect. Compared with controls, SSA attempters reported higher levels of adverse life events and criminal history factors. Being bullied in prison was significant even in multivariate analyses of several factors. While adverse life events were common among the prisoners, bullying, hopelessness, and a parent or sibling’s death were significantly higher among SSA attempters. Bullying was found to be significant even after controlling for other factors in the multivariate analysis.

Another single study investigated the association between geographic mobility and SSAs. Potter et al. (46) found that geographic mobility (changing residence over the past 12 months) among young adults was highly associated with SSAs while only weakly associated with non-SSAs. However, the adjusted odds ratio of this result was relatively low (2.1, with 95% confidence interval of 1.4–3.3). When looking more specifically at the risk factor—recent (in the past 3 months) or three or more relocations over the past 12 months—the OR rises to 6.2 (3.0–13.3). Thus, it seems that recent and frequent geographic mobility are more refined risk factors for SSA.

Although unemployment is a common stressful event often leading to various experiences of psychopathologies, we found only one study (from 1998) that evaluated the link between unemployment and SSAs. Beautrais et al. (27) studied 302 SSA attempters and compared them to 1,028 randomly selected community control subjects. They found that SSA attempters reported higher rates of current unemployment (OR = 4.2) compared with controls. This correlation was similar for males and females. Following adjustment for preexisting childhood, familial, and educational factors, the association between unemployment and risk of SSA decreased, but remained significant (OR = 2.1). Nevertheless, when both preceding family and childhood factors, and psychiatric morbidity were considered, unemployment was not significantly related to risk of SSA. The authors concluded that the link between unemployment and suicidal behavior was to a large extent non-causal, and reflected mutual or correlated factors contributing to risks of both unemployment and suicidal behavior. Any remaining association between unemployment and SA risk appeared to result from the correlation between unemployment and psychiatric disorder.

## Discussion

Most studies regard SAs as a unified phenomenon, while neglecting to consider differences between levels of suicidal behaviors. Disregarding these distinctions impedes the detection of those at high risk for suicide. This is also partly because these studies tend to draw conclusions from findings regarding attempters to suicide completers.

To overcome this obstacle, researchers have developed the SSA strategy, which focuses on individuals who made definite, near-fatal attempts but who did not die from suicide due to intensive medical intervention. This group seems to resemble suicide victims quite closely (14, 20). Therefore, it may provide some insight into the large group of suicide completers whose first attempt is fatal, and who have hitherto been neglected by mental health professionals. More specifically, the SSA approach can provide insight into understanding the personal and interpersonal characteristics, circumstances, and psychological processes leading to SAs. However, to the best of our knowledge, no study has systematically reviewed the empirical data accumulated in this field to date.

In what follows, we would like first to summarize and integrate the main findings of our review. Next, we will highlight methodological limitations akin to the study of SSA.

### Risk Factors for SSA: Toward an Integration of a Model

Drawing on the SSA approach, several projects and studies [e.g., (14, 18, 48, 72)] highlighted important psychological characteristics and variables that can serve as risk factors of SSA. In this paper, we aimed to systematically review the empirical data accumulated thus far regarding the psychological risk factors for severe SAs.

In general, several categories of psychological risk factors and warning signs for SSA were identified: psychopathology and mental pain, interpersonal relationships, impulsivity and aggressiveness, decision making, and several-specific isolated factors, such as negative life events. High levels of psychopathology [e.g., Ref. (14)] and unbearable mental pain were found to be conditions of susceptibility to SSAs, and to play a major role in creating the basis for suicide acts. Specifically, the review findings revealed the unique value of mental illness, particularly the diagnoses of MDD, anxiety disorder, PTSD, anti-social personality disorder, substance abuse/dependence disorders, and psychiatric comorbidity as predictors of SSAs (14, 41, 48). Along with psychopathology, psychological pain [including hopelessness and depression experience (64)] was a distinguishing factor between serious suicide attempters and healthy populations in various studies. In other words, levels of psychopathology and mental pain were associated with an increased risk for SSA compared with controls. These recurring results emphasize that psychopathology and mental pain may serve as vulnerability factors that need to be considered when attempting to predict SSAs in large community populations.

However, when comparing SSA attempters to those with lower severity, psychopathology, and mental pain dimensions alone cannot effectively predict SSAs (38, 52, 57). Nonetheless, several studies found that these dimensions play a critical role in cases where the individual is unable to regulate or adjust the experienced emotional pain (18, 19). In other words, if mental pain is high, its effect on suicide severity depends on other psychological factors that serve as facilitators, or protective factors against SSAs.

The most essential factor that was found to moderate the facilitation of mental pain and should serve as critical risk factor to SSAs was the interpersonal abilities dimension. Several studies emphasized that in instances in which mental pain is combined with an inability to communicate stress, painful feelings remain unaddressed, thereby resulting in more serious forms of suicidal behavior with higher severity levels of the suicide intent’s objective components (18, 37, 38). Put differently, combined with mental pain, interpersonal, and communication difficulties expressed by loneliness, schizoid traits, alexithymia, lack of social support (18), insecure attachment style (39), low-actual social interaction (14), limited help-seeking behaviors (25), and inability to disclose emotions and thoughts (19) interact to facilitate SSAs (18).

Another important psychological risk factor for SSAs is the impaired cognitive control during rule learning, which may explain some of the reasons for choosing highly lethal suicide methods leading in turn, to medically severe SAs. In several studies, individuals who made SSAs were found to be more inclined to delay rewards (31), had poorer conceptual reasoning, and more conceptual errors. Interestingly, SSA attempters were found to decide and choose without considering the cost and implications of their decisions (55). It seems that while some decision-making impairments are associated with suicidal behavior in general, specific difficulties are related to more serious suicide behaviors. These difficulties can be categorized as the lack of ability to calculate costs and gains as a result of one’s behavior, and the reluctance to modify choices when deciding on actions (e.g., willingness to delay rewards and high-punishment regardless of the costs). Thus, these impairments should be carefully considered when attempting to assess the probability for SSAs. It should be noted, however, that the research on decision making and SSAs focuses mainly on elderly populations. No data are available on this relationship among young and young adult participants. Obviously, more research is needed to generalize and refine these results in terms of other SSA attempter populations.

The impulsivity–aggressive dimension was also investigated as a possible risk factor for SSAs. In general, the impulsivity, aggression, and hostility factors were found to differentiate between suicide attempters from non-attempters, but in most studies, both impulsivity and the related constructs failed to account for differences between high- and low-lethality attempters. Thus, it seems that this dimension is a more general risk factor for suicidality than it is a specific factor related to severe SAs. It can be concluded that while characteristics of planning and preparation are important factors related to the seriousness of the attempt, highly lethal acts depend also on circumstantial factors (like availability of means) and are often performed on the spur of the moment with limited planning. Interestingly, in a follow-up study (36), impulsivity, anger-out, and violence were found to have significant positive correlations with medical severity of follow-up SAs among those who made SSAs in the index attempt. Thus, the impulsivity–aggressive dimension does play some role in the potential for higher lethal SAs among subjects with a history of SSAs. One can conclude, therefore, that when a person has made SSAs in the past, the potential for a repeated serious attempt is higher, especially when the decision is impulsive and made on the spur of the moment.

Some studies found that recurrent stressful life events can also serve as SSA risk factors. Among particularly traumatic events, the evidence points to childhood sexual abuse (14, 44), geographic mobility, and unemployment.

**Figure 2:**
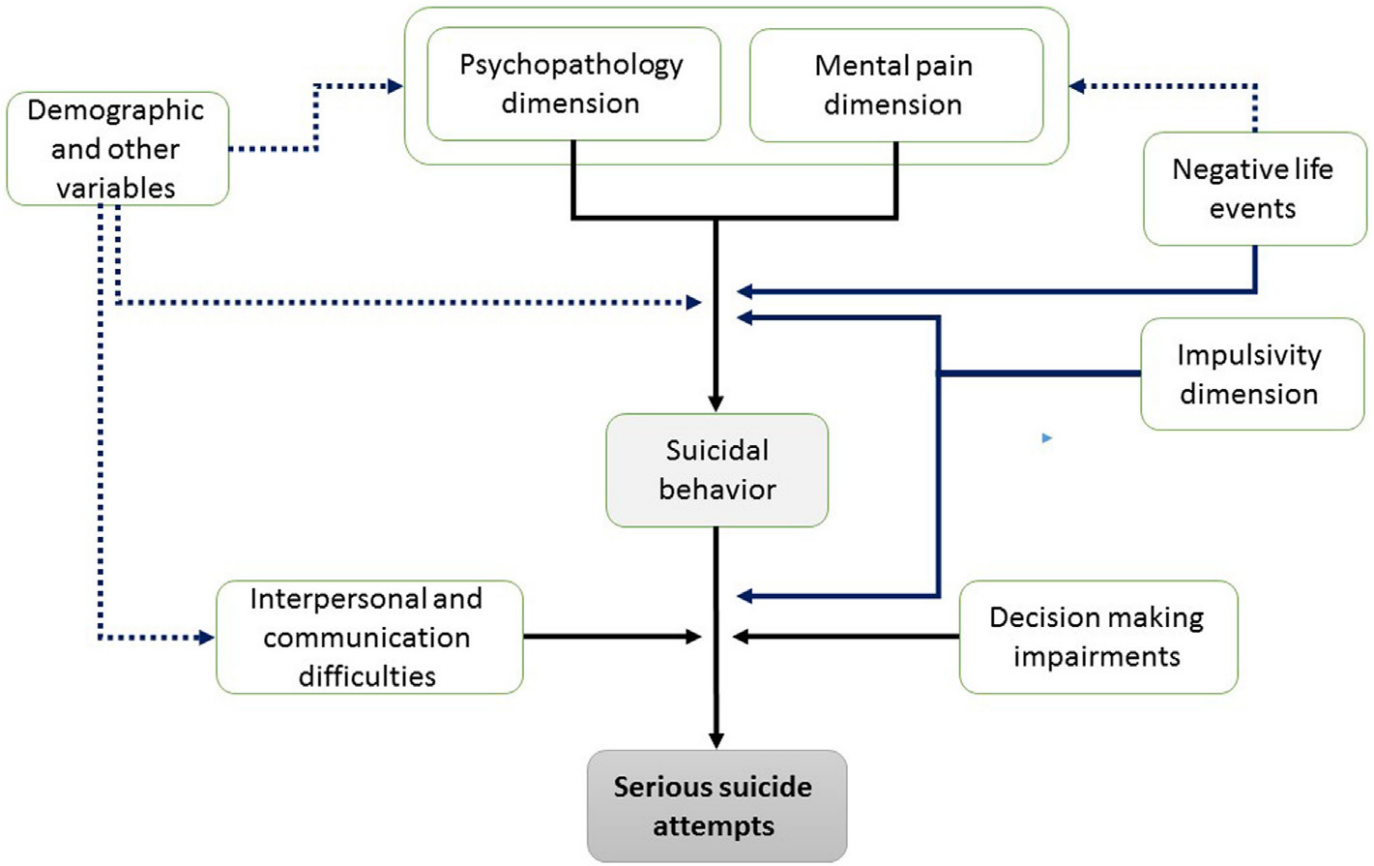
Proposed psychological model of risk factors to serious suicide attempts.

Taking together, and in line with Levi et al. (38) model, we propose an integrative model of psychological risk factors for SSAs. This model contains vulnerability factors, immanent factors, and facilitating factors in three stages of occurrence (see Figure 2). In the first stage of the proposed model, mental pain experience, psychopathology, and drug and substance abuse converge to create an atmosphere of unbearable mental pain, which is similarly manifested among all suicide attempters. Notably, the combination of this personal state with lack of interpersonal and communications abilities, as well as with impairments in decision making and specific negative life events, seems to generate an “impossible situation,” which in turn may provoke an SSA. In other words, the combination of vulnerability factors with the critical risk factors for SSAs in stage 2 (and other demographic factors), render the probability for SSAs profoundly higher. In this devastating state of “impossible situation,” higher levels of impulsivity and aggression may act as facilitators and aggravate suicidality levels to highly lethal SAs.

This systematic review highlights that while personal factors and psychopathology are the main risk factors for SAs in general, the critical elements that facilitate higher levels of dangerous suicidal acts are difficulties in communication and impaired decision making. The insights from these results are twofold: first, in order to accurately assess those at risk for SSA and even more dangerous suicidal acts, it is important to examine the patient’s levels of communication and social connectedness, as well their decision-making processes with emphasis on their lack of cognitive control. Second, these dimensions should be at the crux of all therapy goals. Several therapeutic protocols (e.g., interpersonal psychotherapy), focused on these dimensions are already available.

### Methodological Limitations to the Study of SSA

One of the main complications associated with the study of SSA is participant recruitment. The definition of SSA entails the understanding that the attempter was saved merely by chance and/or due to intensive medical intervention. This explains the scarce data and small SSA samples. Moreover, several studies reviewed here [e.g., Ref. (31, 55)] restricted assessment to specific groups (e.g., elderly people), thereby limiting the ability to generalize conclusions.

Another important drawback lies with the definition of, and criteria for, SSA. Most studies employ the terminology of severe suicidality; however, given that the criteria for SSA are highly variable, in practice researchers often apply non-stringent criteria (e.g., lethality rating scale >3). This decreases the ability to infer data regarding SSAs, whereas the reliance on a strict and consistent definition makes it possible, to a large extent (13, 14), to infer information from SSAs, thereby increasing the ability to identify the risk factors for suicide.

This study stems from the awareness that near lethal attempters are a unique group, distinct from non-SSA attempters. Nevertheless, the literature survey revealed that only a few studies compared SSAs to non-SSAs. A large portion of the studies reviewed here compared SSAs to non-suicidal psychiatric and/or general population controls rather than use non-serious suicide attempters as a comparison group. This weakens the ability to draw conclusions about the SSAs group.

An important limitation of the SSAs strategy is related to the use of medical lethality as a central criterion. While most studies adopted this criterion [e.g., Ref. (18, 19)], its main drawback is that it does not take into consideration that the outcome of any suicide method is heavily influenced by chance factors and circumstances. Put differently, while the post-SA physical condition is an objective criterion with several advantages, it may be greatly influenced by the method’s availability or imprecise knowledge of the employed method’s lethality (73). For example, the extent and nature of physical harm aligned with medication overdoses is significantly contingent on the type of medication: SSRI antidepressants cause limited physical damage, whereas use of tricyclic drugs will most probably warrant intensive hospital care. Considering, however, that the choice of medication is usually limited to the attempter’s prescription drugs—of which they are ignorant as to potentially lethal effects—it is not closely related to the actual seriousness of the intent to die. Thus, in future studies it is important to assess actual suicidal intent and take it into account when defining SSAs that closely resemble actual suicides (20). Incorporating an intent factor can also help refine the definition for SSA as limited to particularly severe SAs (21).

Lastly, a broad limitation is the use of retrospective self-report measures for collecting data on the majority of psychological factors mentioned in this review. These measures may introduce a familiar range of biases triggered by factors such as mood dependent recall, forgetfulness, cathartic effect, and social desirability.

### Implications for Research and Treatment

Serious suicide attempts are a valid proxy for completed suicides, and therefore, the variables that predict severe suicidal behavior should be at the forefront of the research and clinical efforts when assessing risk groups for suicide. Future studies need to consider the importance of strict and consistent definition criteria for SSAs to improve research strategies, assessment, and preventive efforts. This review also highlights the importance for mental health professionals and clinicians to further assess patients who present psychopathology and distress. Those patients should be asked about their support circles, and ability to recruit the environment for help. As decision-making processes have been found to increase the risk of serious medical suicidal behavior when distress is in the background, it is important to evaluate the patient’s ability to make flexible decisions, to calculate the consequences of his behavior (e.g., suicide), and to modify his choices accordingly.

## Conclusion

Overall, our findings provide a foundation for future research regarding the combined role of psychopathology, mental pain, communication difficulties, decision-making patterns, and facets of impulsivity and aggression, in the occurrence of near-lethal SAs. Moreover, our review suggests that the developmental paths culminating in suicidal behavior are heterogeneous, and that different mechanisms or processes are more likely in play for those who commit suicide as opposed to those who attempt non-lethal suicidal attempts. Regardless of the limited scope of research and available data, it is important that health care professionals consider these risk factors when assessing the risk of future severe SAs and suicide.

## Author Contributions

We were both involved in the initiation of the subject, searching the web, categorizing and integrating the data, and in writing the paper.

## Conflict of Interest Statement

The authors declare that the research was conducted in the absence of any commercial or financial relationships that could be construed as a potential conflict of interest.
